# Soft Materials for Photoelectrochemical Fuel Production

**DOI:** 10.1021/acsenergylett.3c01782

**Published:** 2023-11-20

**Authors:** Erin L. Ratcliff, Zhiting Chen, Casey M. Davis, Eui Hyun Suh, Michael F. Toney, Neal R. Armstrong, Obadiah G. Reid, Ann L. Greenaway

**Affiliations:** † Department of Chemical and Environmental Engineering, University of Arizona, Tucson, Arizona 85721, United States; ‡ Department of Chemistry, University of Colorado Boulder, Boulder, Colorado 80309, United States; § Materials Science and Engineering Program, Department of Chemical and Biological Engineering, University of Colorado Boulder, Boulder, Colorado 80309, United States; ∥ Renewable and Sustainable Energy Institute, University of Colorado Boulder, Boulder, Colorado 80303, United States; ⊥ Department of Chemistry and Biochemistry, University of Arizona, Tucson, Arizona 85721, United States; # Materials, Chemistry, and Computational Science Directorate, National Renewable Energy Laboratory, Golden, Colorado 80401, United States

## Abstract

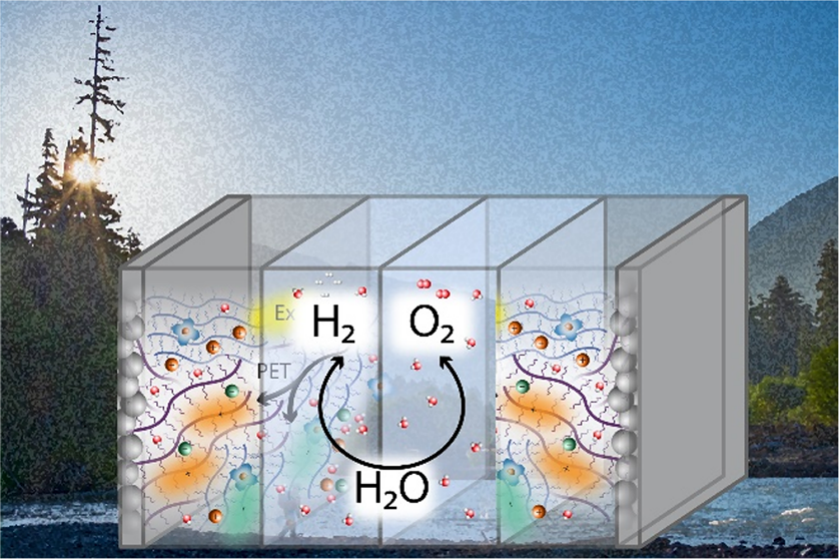

Polymer semiconductors are fascinating materials that could enable delivery of chemical fuels from water and sunlight, offering several potential advantages over their inorganic counterparts. These include extensive synthetic tunability of optoelectronic and redox properties and unique opportunities to tailor catalytic sites via chemical control over the nanoenvironment. Added to this is proven functionality of polymer semiconductors in solar cells, low-cost processability, and potential for large-area scalability. Herein we discuss recent progress on soft photoelectrochemical systems and define three critical knowledge gaps that must be closed for these materials to reach their full potential. We must (1) understand the influence of electrolyte penetration on photoinduced charge separation, transport, and recombination, (2) learn to exploit the swollen polymer/electrolyte interphase to drive selective fuel formation, and (3) establish co-design criteria for soft materials that sustain function in the face of harsh chemical challenges. Achieving these formidable goals would enable tailorable systems for driving photoelectrochemical fuel production at scale.

Photon-to-electron-to-molecule energy conversion–termed solar fuel generation–will support the U.S. goal of net-zero CO_2_ emissions by 2050^1−5^ and improve long-term storage of intermittently generated green energy.^6−8^ Such a large scale effort will require investment in a number of technologies. One emerging strategy is to use highly scalable, printable, soft materials as semiconductor photoelectrodes for photoelectrochemical (PEC) devices, which offer opportunities to further enhance the tailorability of local environments that drive multielectron, multiproton redox reactions. Herein, we present a perspective on the basic energy sciences of polymer-based semiconductors and the way that these “soft” semiconductors may enable tunable and durable devices for solar fuel generation. Simultaneously, we address how basic energy science research can and should advance societal aspirations of a just energy future (see Box 1: Energy Justice).

Box 1: Energy Justice *Energy justice* is the goal of achieving equity in the energy system while remediating burdens on those who have been disproportionately harmed by the energy system.^16,17^ Energy justice likely cannot be achieved by deploying a single technology and ultimately will include, among other efforts, policy initiatives at many levels. A timeline highlighting some energy justice initiatives is included in Figure 2e. Here we emphasize a less-considered factor: the need to implement energy justice approaches at the earliest stages of energy research in chemistry and materials science.

Energy justice *can* and should be considered a factor in basic energy research, analogous to guiding factors of cost and scalability. As the basic science of soft PEC systems develops, researchers pursuing this work can make fundamental changes to experimental approaches before decisions leading to unjust outcomes become accepted practices at higher stages of technological readiness.^18^ For instance, technoeconomic studies^10,19,20^ have emphasized the printability and scalability of polymer semiconductors, but hidden process costs such as waste from toxic solvents^21,22^ are ripe for improvement even at bench scale. Basic energy research often does not consider the externalities associated with material production, but a range of literature shows the negative impacts of polymer production,^23,24^ which should be considered in the development of these new technologies even as soft semiconductors offer improved cost and scalability over hard PEC systems. Researchers can also publish articles open-access to increase exposure in the general community and opportunities for feedback to the scientific process, even in early stage work.^18^ These suggestions are among many actions that researchers can take while pursuing fundamental science to ensure that new energy technologies are deployed in a way that is sustainable and that mitigates or reverses the harms from the existing energy system.

A defining characteristic of soft semiconductors in the context of photoelectrochemistry is that the electrolyte can—at high volumetric electrochemical doping—penetrate deeply into the polymer semiconductor, changing its structure and properties profoundly relative to the “dry” (as-deposited) material. We adopt the terminology of *interphase* to broadly represent the multilength scale phenomena that occur with polymer-electrolyte-solvent interactions, with a partial analogy to the solid-electrolyte interphase (SEI) used in conventional battery descriptions. The unique characteristics of this “interphase” of a polymer-based photoelectrode are illustrated in Figure 1, which diagrams processes that are ubiquitous to both photoelectrochemical and electrochemical applications of soft materials. Photochemistry begins with light absorption on a polymer chain and subsequent charge transfer at a blended donor–acceptor heterojunction formed from two different polymers. Energy offsets between the electron donor and acceptor polymers drive dissociation of high binding energy excitons in a process that is in part controlled by the local polymer chemical and physical structure and interphase dielectric environment, which we jointly refer to as the *nanoenvironment*. Charges subsequently migrate to catalytically active sites through chemical potential gradients. The large molar absorptivity across the solar spectrum of organic chromophores enables active layers on the order of hundreds of nanometers thick, which suggests the interphase can make up the entire system while encompassing a range of local nanoenvironments. Ions and solvent from the electrolyte can insert into the polymer layer and the resulting swelling and mixed electrical-ion conduction may promote or impede (photo)electrochemical charge transfer processes. Critically, molecular-based systems do not exhibit charge-trapping surface states due to dangling bonds but rather are comprised of narrower distributions of molecular-orbital-based electronic states with energies that can be easily tuned synthetically by altering the molecular building blocks of the polymer. The many components of a soft PEC system—e.g., donor and acceptor polymers, catalysts, and electrolyte—will ultimately function as a whole, and must be *co-designed*, rather than individually examined in sequence. The temporal evolution of the interphase and local nanoenvironments under PEC operation require that each component be treated as part of the whole, ultimately enabling design based on the realized properties of polymers under operation for more stable and durable systems.

**Figure 1:**
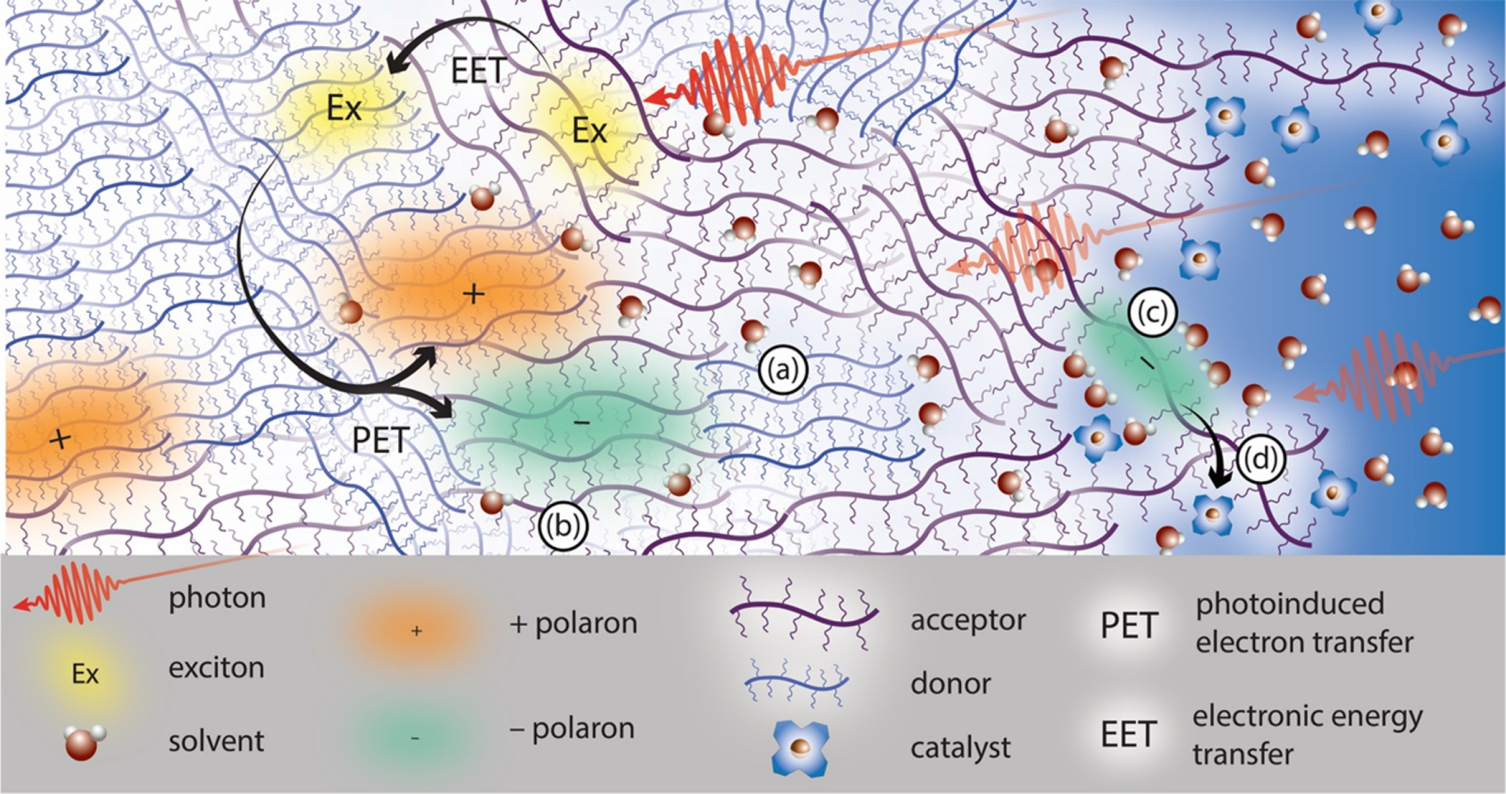
Schematic illustration of the polymer-electrolyte interphase, a term specifically chosen to describe the longer length scale phenomena that occurs with polymer-electrolyte interaction. Illustration shows processes occurring in a two-component polymer blends (donor and acceptor) and polymer-electrolyte system that can generate solar fuels, including light absorption to form an exciton, excitation energy transfer (EET) and exciton diffusion, photoinduced electron transfer (PET), and diffusion of charges to the edge of the polymer. The major species present are defined in the legend, with electrolyte ions omitted for simplicity. The scientific questions noted in the text connect to (a), (b), (c), and (d). Scientific questions are illustrated at several points: (a) To what extent do solvents swell the bulk of the film, and what impact does this have on structure and electronic properties? (b) Does the presence of polar solvent molecules modify the energy landscape and the coupled dynamics of charge separation? (c) Does solvent stabilization of charges at the interface prolong their lifetime and ability to do electrochemical work, possibly through surface attached catalysts targeted to specific reactions (d)?

## Scope of this Perspective

While many of these unique characteristics are factors understood by comparison to other organic semiconductor-based technologies, PEC applications are under-explored and many questions remain. Major knowledge gaps are highlighted in Figure 1: (a) To what extent do solvents swell the bulk of the film, and what is the impact on structure and electronic properties? (b) Does the presence of polar solvent molecules modify the energy landscape and the coupled dynamics of charge generation, transport within the film, or transfer out of the film? (c) Does solvent stabilization of charges at the interphase prolong charge lifetime and ability to do electrochemical work, possibly through surface attached catalysts targeted to specific reactions? (d) How do extended exposure and cycling in the presence of operational stressors (e.g., light, oxygen, harsh solvent conditions) impact charge transport, transfer, and durability, and can the components of a soft polymer system be co-designed to enable continued performance under those conditions? Below we identify the emergence of these key knowledge gaps, considering a historical perspective of the origins of the field, a materials-level comparison between “soft” polymer semiconductors and “hard” inorganic semiconductors, and a recent summary of the emerging field that combines electrochemistry and soft materials. We further emphasize that many of these questions have applications that transcend the photoelectrochemistry of solar fuels and if resolved, have fundamental implications across a number of energy conversion and storage platforms. We focus here on PEC water splitting by soft photoelectrodes, but the questions raised are equally relevant to soft polymer PEC carbon dioxide reduction, which is a rapidly expanding field in its own right.^9−11^ While some issues highlighted here are also relevant to the emerging field of covalent organic frameworks (COFs) for PEC applications, we focus here on π-conjugated semiconducting polymers, and refer readers to related work focused on COFs.^12−15^

## Existing Technologies Relevant to Soft Photoelectrochemical Systems

**Figure 2:**
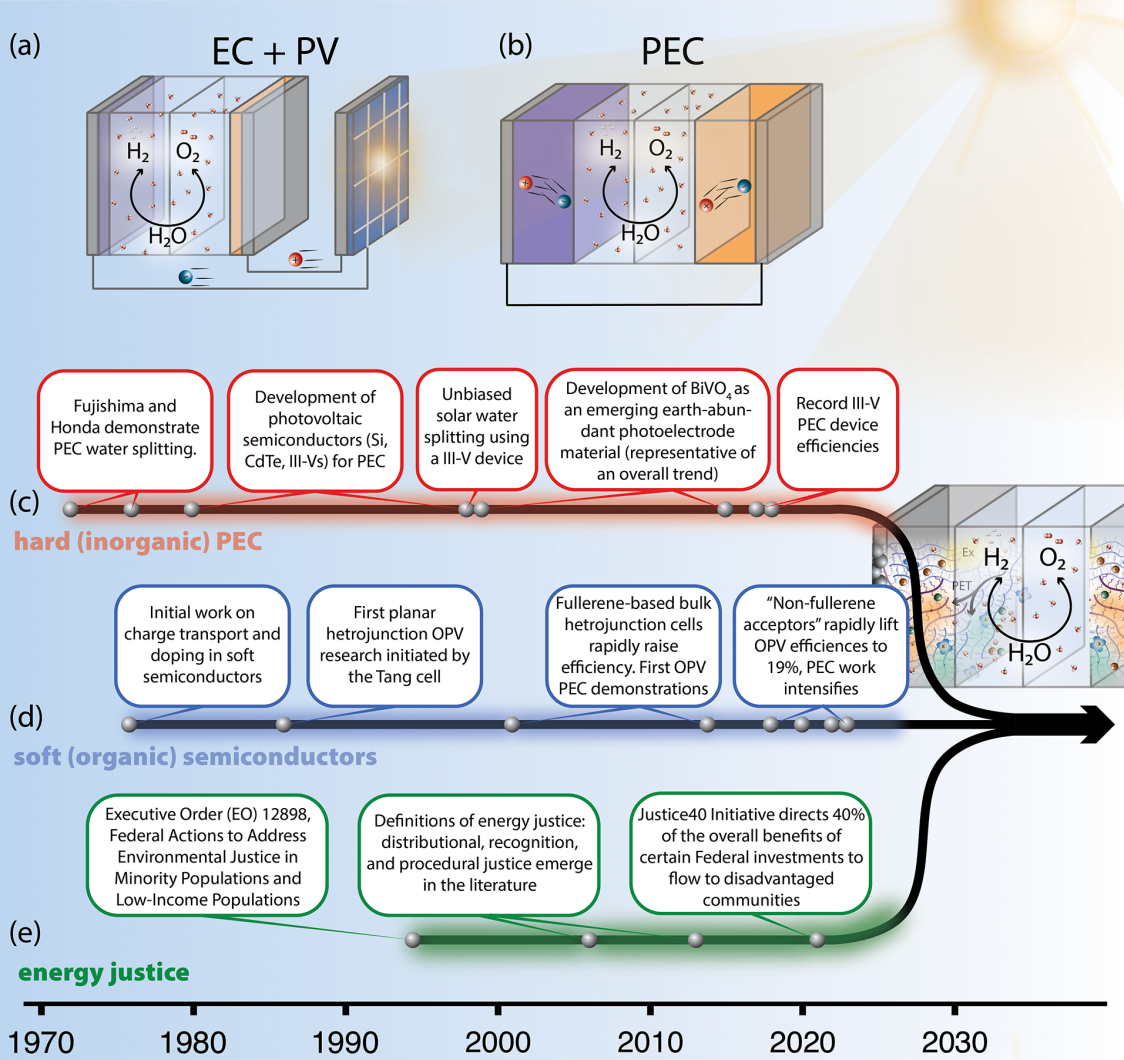
Schematic illustrations of (a) existing technology of photovoltaic electrolysis (PV-EC) and (b) the long-term goal of photoelectrochemical devices (PEC), as well as timelines of major developments in (c) hard (inorganic) PEC devices (referring to refs (37−42)); (d) soft (organic) semiconductors and related technologies (referring to refs (43−49)), and (e) highlighted considerations around energy justice in basic energy science(referring to refs (17,50−52)).

Historically, there are two methods of solar H_2_ generation: photovoltaic-driven electrolysis (PV-EC) and photoelectrochemistry (PEC). In PV-EC (Figure 2a), photons excite electrons in photovoltaic panels; the electrochemical potential of those electrons provides the voltage needed for catalysts/electrolyzers to split water. PV-EC technologies are already commercialized but suffer from substantial efficiency losses^25−30^ and high materials and maintenance costs from integrating two disparate systems.^31^ Alternatively, in PEC systems (Figure 2b), photons excite electrons in semiconductors directly integrated with catalysts to drive the water splitting reactions. The close integration of semiconductor, catalyst, and electrolyte in PEC presents opportunities to tailor chemical functionality at molecular length scales through co-design to ensure efficient capture of all generated electrons and long-term stable operation.^30^ PEC has yet to be commercialized, but a developed technology may reduce cost^31,32^ and improve efficiency over PV-EC,^31^ and deployment could enable off-grid or remote fuel production from a single device package.^27,33^ Beyond H_2_ as a solar fuel, the molecular tailorability of PEC provides broad technological opportunities, including generation of other solar fuels, such as via CO_2_ valorization,^30^ and connections to biological sensing and healthcare.^34−36^

An understanding of the unique challenges in soft photoelectrochemical systems emerges from the historical trajectory of PEC research. A brief timeline of developments in “hard” inorganic semiconductor PEC is presented in Figure 2c. The first demonstrations of water splitting via PEC used wide bandgap metal oxide photoanodes and noble metal cathodes,^37,38,53^ but quickly expanded to photoelectrodes based on Si and III-Vs, among others.^53^ Working models of inorganic semiconductor PEC behavior emerged from concepts of band bending and flat band potentials.^53^ Significant efforts have been devoted to discovering electrochemically stable, catalytically active, hard semiconductors with moderate bandgaps (>1.6 eV),^30^ with a substantial recent focus on metal oxides.^40,54,55^ However, challenges of stability and scalability remain.^30^ Devices based on III–V semiconductors have the highest PEC efficiencies^41,42^ but require intensive tailoring of compositions, structures, contacts, and protective layers, all of which increase cost at scale.^30^ Additionally, water splitting reactions are most efficient at high or low pH, where many hard semiconductors are unstable;^56,57^ many of those which are stable are less-developed, resulting in a trade-off between efficiency and durability.^58^ Finally, electrocatalyst integration is often necessary to increase PEC reaction rates. Although the field is targeting earth-abundant catalysts^59,60^ and/or selective molecular catalysts,^61,62^ long-term stable integration of any catalyst on a hard semiconductor with good charge transfer is a persistent problem.^63−65^

The field of organic electronics developed in parallel (timeline in Figure 2d). These molecular and polymeric semiconductors are highly scalable to large areas, and generally lower cost by area than hard semiconductors due to their low temperature solution-phase synthesis and processing.^19,20^ Perhaps most importantly, organic semiconductors offer synthetic tunability in their optical and redox properties in ways which are inaccessible to hard materials. Foundational work for organic PEC comes from studies of doping of conductive polymers^43,44^ and the photophysics of diode behaviors which necessitate energetic offsets in ionization potentials and electron affinities to drive charge transfer.^45^ In organic photovoltaics (OPV), the early planar structures were replaced by nanometer-scale blended heterojunctions (BHJs) of donors and acceptors which require exquisite control of microstructure to simultaneously optimize charge transfer and charge transport phenomena. The chemical nature of these soft materials has allowed for intricate spectroscopic interrogations of excitonic and polaronic species, which have supported improvements in OPV^46,66−69^ and commercialized organic light-emitting diode (OLED) technologies.

## Challenges and Opportunities

**Figure 3:**
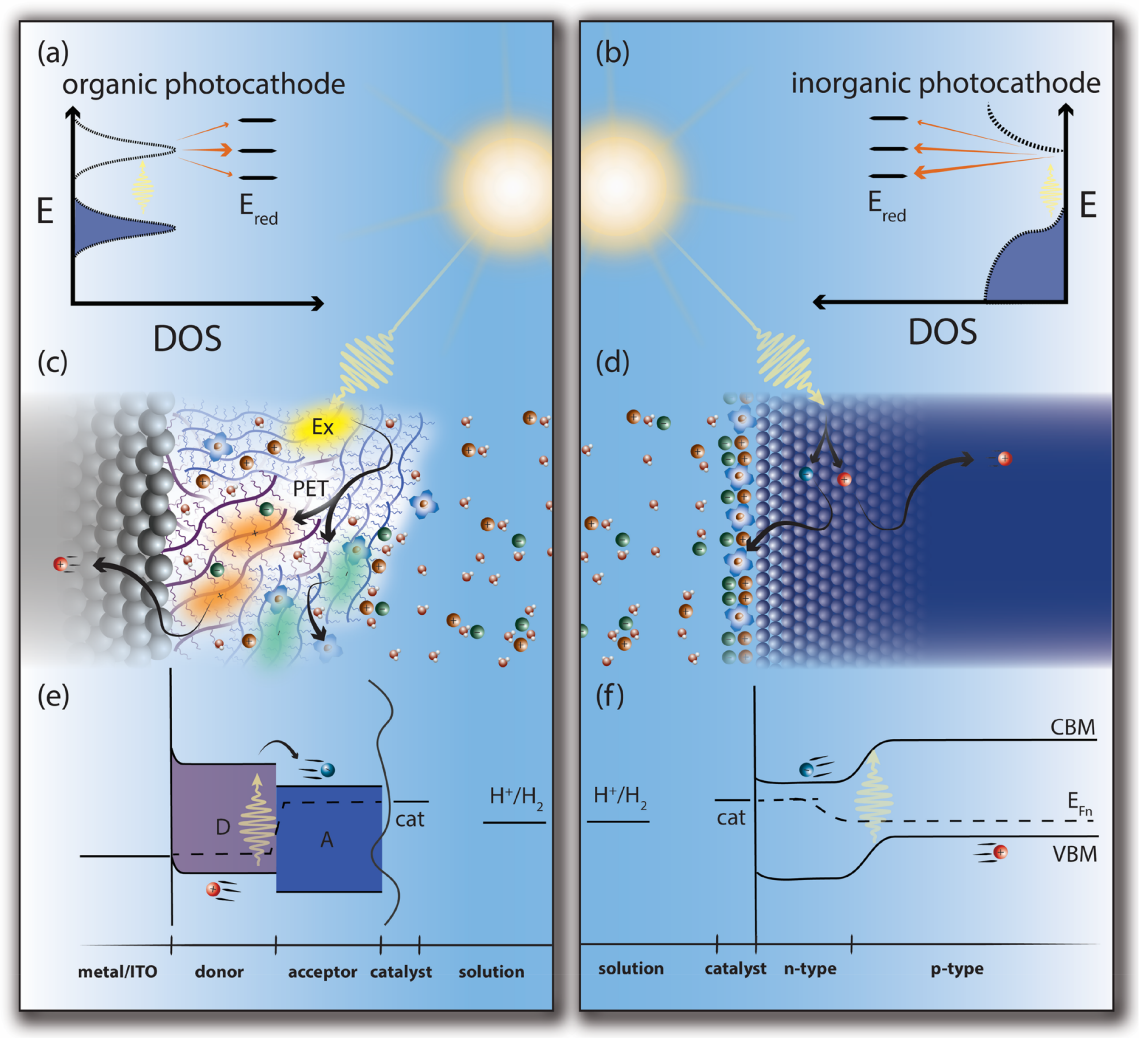
Comparisons between soft (organic) and hard (inorganic) semiconductors utilized as photocathodes for HER to illustrate major differences. (a, b) Generalized density of states (DOS) of the two classes, illustrating the possible reaction tailoring based on orbital overlap for soft systems compared to hard systems. (c, d) Illustrations of interactions between electrolytes (solvent, solvated reactants, and ions) and surfaces of soft and hard systems. (e, f) Illustrative band diagrams for soft and hard PEC systems, assuming a buried junction hard photocathode. Acronyms used are valence band minimum (VBM), conduction band maximum (CBM), electron quasi-Fermi level (E_Fn_), catalyst (cat), electron donor polymer (D), electron acceptor polymer (A), indium tin oxide (ITO), exciton (Ex), photoinduced electron transfer (PET).

Currently, single-junction inorganic photocathodes exhibit half-cell solar-to-hydrogen (STH) conversions of 10–13%, while the highest reported organic half-cell photocathode STH is ∼3.78%.^70,71^ Examining the differences between soft and hard PEC systems (illustrated in Figure 3) provides a useful framework to understand critical knowledge gaps in the fundamental behavior of soft semiconductors for PEC. In a soft semiconductor system, charge transfer has been suggested to occur from an electrochemically active density of states^72^ rather than from a band edge (Figure 3a,b). Electrical charge carriers in unique nanoenvironments within the interphase region can have different strengths of oxidizing or reducing power; understanding and controlling these nanoenvironments provides a strategic opportunity for co-design of functionalities. For example, inorganic semiconductors must be doped during initial synthesis or subsequent processing to generate a rectifying contact with an electrolyte—i.e., sufficient electric field exists for band-bending to force charge carriers to the interface.^73^ The temporally static, nanoscale-sharp boundary of hard semiconductor/electrolyte interfaces is in distinct contrast to the polymer-electrolyte interphase, which is substantially wider than a few atoms or even individual polymer chains,^74^ incorporates ions from the electrolyte,^75^ and changes with time, carrier density, and electrolyte incorporation in situ;^76^ these interactions are illustrated in Figure 3c,d. The multiscale heterogeneity of the polymer surface controls the concentration and reactivity of charge-transfer sites for soft PEC systems,^77^ so redox reactions may proceed at dramatically different rates and change over time with electrolyte insertion (Figure 3e). This offers an opportunity to tune multielectron reactions thru kinetic pathways. In contrast, the band edge position of hard semiconductors dictates their ability to generate a charge carrier that is sufficiently energetic to drive a reaction, and charge transfer may depend on a surface dipole,^73^ surface states, or appended catalysts^59^ but is generally fast (Figure 3f).^78^

Unlike hard semiconductor systems, where surface states and bound catalysts may modify charge transfer and redox rates but the bulk of the material remains unaffected, charge transport and transfer in soft semiconductors are highly dependent on the local micro- or nanoenvironment, which includes effective interchain wave function overlap that differs in crystalline and amorphous regions^76^ and thus alters charge mobility,^79^ but are rarely considered in the design or analysis of polymer PEC systems. For instance, these fundamental changes *must* influence the capacitance at the polymer|electrolyte interphase, but are seldom,^80^ if ever, incorporated into the circuit models used to measure the voltage-dependent capacitance. Electrostatic interactions with outer-sphere redox molecules, such as methyl viologen, can be harnessed to enable molecule release from a surface after the redox reaction has occurred,^81^ but can equally hinder such release. If redox reactions occur at more extreme potentials than electrochemical doping and dopants are easy to remove, then dopant loss can out-compete charge transfer to a redox molecule.^75^ All of these factors are further complicated by electrode cycling and the duration of operation, and illustrate the broad scope of scientific development needed for soft PEC systems.

We now turn our attention to recent demonstrations of soft semiconductors in PEC applications. Many recent examples replace the top contact of OPV devices with an electrolyte/redox couple and consider half-cell performance.^82−85^ Demonstrations include both photoanodes^10,86^ and photocathodes,^10,87,88^ with complementary applications to photoelectrochemical processes of biological relevance such as artificial retina^89,90^ and the functions of living cells.^91,92^ Despite this substantial record in the literature, remarkably little is known about what photophysical changes occur when soft polymer semiconductors are exposed to polar electrolyte environments and how these properties couple to changes in nanostructure, charge transport, and charge transfer to determine material- or device-level functionality. Recent focus has targeted buried junction devices, where the photoactive organic layer is not in direct contact with the electrolyte.^49,83,87,93−96^ Rather, a charge-selective layer is interfaced between the semiconductor and the electrolyte to limit recombination, protect the organic semiconductor layer, and/or reduce the need to tailor the frontier orbitals of the organic semiconductor to the electrochemical reaction.^87^ However, such layers can quickly fail^83^ (as they do even in hard PEC systems^58^) and the electrolyte will contact the organic semiconductor. Thus, understanding the chemical and physical consequences of the polymer-electrolyte interactions is critical. Moreover, the buried-junction approach precludes exploitation of the molecular tunabilty inherent to soft materials to control energy levels and integration with catalysts, shifting the problem to the challenge of catalysts on metal oxide supports.^59^ We postulate that the distributed interphase created when a conjugated polymer becomes electrochemically doped and swollen with electrolyte is likely to afford new scientific and technological possibilities not available through the buried junction approach.

Durability is increasingly being considered as a factor in the recent work on both hard and soft PEC systems, and as our understanding of changes to charge transport and transfer improves, new systems must be designed with retention of function in mind. Although PEC cells based on conjugated polymers to date have operational lifetimes far from that needed for practical lifespans of solar fuel generation (>10 years),^31^ there are emerging guidelines to design robust and durable interfaces. Conjugated polymers are potentially advantageous to withstand acidic and alkaline electrolyte media if durability is properly understood; their chemically tunable structures could enable chemical and structural resiliency to external stresses from the harsh electrochemical environment.^97−99^ Developments in other soft, polymer-based technology platforms (e.g., OPVs, OECTs, etc.) provide insight into the effects of doping from acidic or alkaline electrolyte ingress,^100,101^ electrochemical bias,^102^ light (particularly UV),^103^ and oxygen^104^ on conjugated polymers, although very few studies exist that explicitly consider multistress pathways such as those that soft PEC devices will experience during operation.

Several stress factors impacting durability have recently been explored experimentally, often with conflicting results which call for further investigation. For instance, charge accumulation in the polymer film rather than successful transfer across the interphase may degrade PEC performance, as shown for the accumulation of photogenerated electrons over the order of 100 nC cm^–2^ chemically degrading BHJ photocathodes performing Eu^3+^ reduction.^83^ However, other work suggests negligible physical changes to BHJ polymers after device operation, using optical and Raman spectroscopy, possibly indicating a different mechanism of degradation or that degradation is limited to the interphase.^105,106^ Another factor is the possibility of small molecule acceptors, which enable higher performance OPVs than polymer acceptors, but which are more soluble, possibly causing chemical and microstructural instabilities that can reduce durability.^107,108^ For example, removal of small molecule acceptors improves the underwater stability of OPV devices.^107^ Other work shows that a ternary BHJ with both fullerene and nonfullerene acceptor molecules (PC_71_BM and ITIC) can *enhance* durability and result in very high PEC performance, albeit in a device protected from electrolyte ingress by a carbon plate.^96^ Another issue is electrolyte swelling-driven delamination of BHJ layers from the underlying electrode during PEC operation. Porous oxide electron transport layers^109,110^ under BHJ layers have been demonstrated to enhance electrode adhesion for photoanodes. Side-chain engineering–such as changes from alkyl to polar side chains in random copolymers—may hinder delamination during electrochemical cycles. While this is not an exhaustive list of factors to be considered in soft PEC, it illuminates the need for fundamental exploration to improve understanding of such systems in operation, which should then enable the development of design rules for improved integration of different polymer types, etc. We emphasize herein that advanced technique development, afforded by the number of in situ spectroscopic signatures for soft materials, will be critical to understanding the transformation of polymer semiconductors under these types of stresses.^111^

Closely related to soft PEC, the area of organic colloidal nanoparticle photocatalysts suspended in water has recently received attention^84^ after substantial hydrogen evolution was demonstrated using colloidal nanoparticles decorated with Pt catalyst.^112^ Like many soft PEC devices, these complex nanostructures include an internal heterojunction that splits neutral excited states into free electrons and holes. These nanoparticle systems demonstrate that polymer semiconductors decorated with catalysts can perform inner-sphere redox reactions such as HER without a protective oxide, but fundamental questions remain. Charge transfer mechanisms are difficult to understand on soft polymer systems because the fundamental behavior of the polymer itself is not understood before it is further complicated, e.g., with the formation of heterojunctions or the incorporation of catalysts, which makes improvement difficult.^113^ As in OPV, the formation of polymer heterojunctions alters overall morphology,^112,114^ which will be further modified with interphase formation, and there is little direct insight into the photophysical consequences of these changes. Subsequent reports^115,116^ agree that a large fraction of the charge carrier population in these soft nanoparticles have a remarkably long, millisecond scale lifetime, but that the overall yield, or possibly the mobility, of free charges within the particles is substantially reduced relative to bulk blends of the same materials.^116^ Whether the long-lived but apparently trapped, charges in these particles are the species that actually participate in HER remains to be seen.

Another opportunity to tailor polymer-electrolyte interactions and nanoenvironments relevant to soft PEC is illustrated by the recently explained correlation between photocatalytic activity and Sulphone functional substituents on the polymer backbone.^117,118^ Linear polyfluorene-based copolymers were synthesized with varying amounts of a Sulphone-bearing unit, and the Sulphone content correlated strongly with HER rate and quantum yield, independent of residual Pd catalyst loading.^119^ The difference could not be explained by shifts in excited state lifetime or the energetic driving force for HER, implicating a specific chemical role of Sulphone groups in mediating the reaction. This behavior was explained through a combination of spectroscopic investigations and quantum chemical modeling of the interface.^117^ The hydrophilicity of the Sulphone groups attracts sufficient water to substantially modify the local dielectric constant at nanometer length scales (i.e., the nanoenvironment) and subsequently the energetics of electron transfer, allowing faster oxidation of the sacrificial reductant (triethyl amine) molecules. Moreover, the Sulphone groups appeared to also mediate electron transfer to the cocatalyst Pd particles, though the mechanism was not explained.^117^ The opportunity to modulate the local dielectric constant is an elegantly simple statement that may form an important design criterion for specifically designing reactive sites within conjugated polymer-electrolyte interphases for PEC: a local dielectric constant in these systems can be engineered through chemical modifications of the polymer to drive specific reactions at specified locations.

## Summary and Outlook

**Figure 4:**
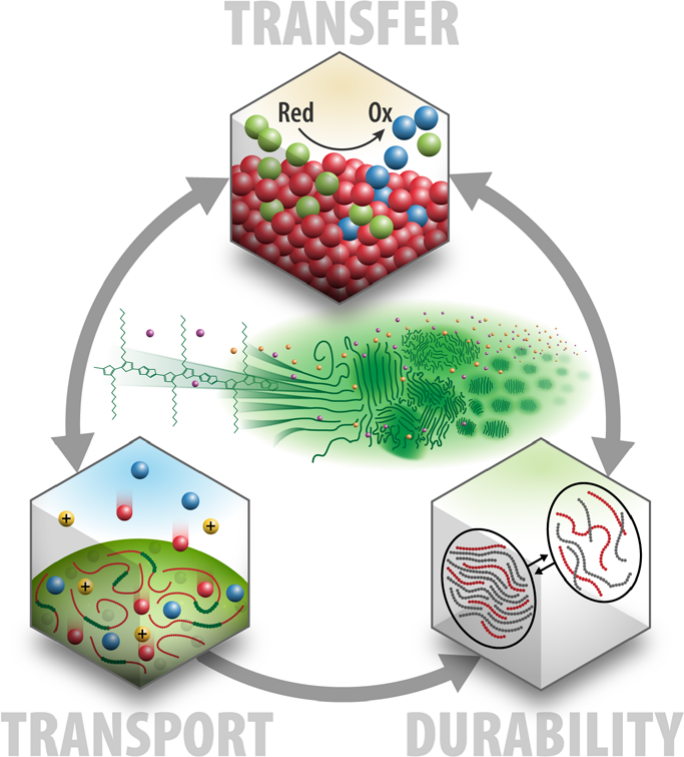
Advancement of the soft photoelectrochemical systems for solar fuels necessitates a multifaceted approach targeting detailed understanding at the molecular level of charge transfer, charge transport and durability to control energy and matter across various spatiotemporal scales. Illustration by Al Hicks, NREL.

In summary, we have detailed many of the advances and new observations in the area of soft photoelectrochemical systems with a focus on charge transfer, charge transport, and durability, as shown in Figure 4. Additionally, we have described the importance of careful scientific decisions during development of this technology to ensure energy justice in a deployed product. The role of the electrolyte—which includes solvent, charge compensating ions, and redox active species—is critical in how it may reorder a donor/acceptor interface, interact with charge transfer states, and alter charge transport. Whether one subscribes to the view that it is disorder, microelectrostatics, charge delocalization, or the long-range tunneling that enables free charge generation in OPV devices, changes in the molecular level structure caused by solvent penetration will be vital to characterize, understand, and ultimately manipulate for soft PEC devices. Local intermolecular order of *at least* the donor or the acceptor is certainly crucial to the generation of free charge. Fortunately, order can increase with modest degrees of electrochemical doping in conjugated polymers, as the nanoenvironment is altered by solvent and ion ingress. Although these factors are not yet well-understood, each provides an opportunity to discretely control (photo)electrochemical charge transfer. Control of charge-transfer reactivity for polymer PEC may be achievable if the local environment of a polymer semiconductor/electrolyte interface can be tuned from the molecular- to mesoscale to promote selective redox pathways. The ability to achieve this will require (1) the development of models describing electric fields and the motion of charges in the polymer/electrolyte interphase; (2) investigation of mechanisms of outer-sphere charge transfer to elucidate rate-limiting factors within the polymer; and (3) application of these structure–property relationships to the mechanisms of inner-sphere redox reactions at polymer|electrolyte interphases.
